# Potential mechanisms underlying inhibition of xenograft lung cancer models by kaempferol: modulation of gut microbiota in activating immune cell function

**DOI:** 10.7150/jca.88038

**Published:** 2024-01-15

**Authors:** Maoying Guan, Weijie Xu, Haoran Bai, Zixiang Geng, Zhihua Yu, Hegen Li, Te Liu

**Affiliations:** 1 Department of Oncology, Longhua Hospital, Shanghai University of Traditional Chinese Medicine, Shanghai 200032, China.; 2 Shanghai Geriatric Institute of Chinese Medicine, Shanghai University of Traditional Chinese Medicine, Shanghai 200032, China.

**Keywords:** kaempferol, lung cancer, gut microbiota, immunity

## Abstract

**Context:** As a flavonoid compound, kaempferol has great potential in anti-lung cancer therapy, but the mechanism of its therapeutic effect needs further exploration.

**Objective:** To explore the therapeutic effect of kaempferol on lung cancer, as well as its capability to regulate the gut microbiota and stimulate immune function.

Materials & methods: Twenty-four BALB/c mice were divided into four groups. The first two groups, consisting of 12 normal mice, were administered either PBS or Kaempferol (Kaem) via gavage. The remaining 12 mice, which were subcutaneously inoculated with Lewis Lung Carcinoma (LLC) cells, were similarly divided and subjected to the same treatments respectively. The inhibitory effect of kaempferol on xenograft lung cancer models was explored with *in vivo* experiments, the diversity of gut microbiota was investigated by 16S rDNA sequencing, and the treatment effect on immune cells was quantified using flow cytometry.

**Results:** Kaempferol exerted a significant inhibitory effect on xenograft lung cancer models *in vivo*. It effectively inhibited the proliferation of LLC cells and significantly activated cytotoxic T cells, natural killer cells, and other immune cells in mice. 16S rRNA sequencing of fecal samples from tumor-bearing mice treated with kaempferol showed a significant increase in the abundances of potentially advantageous microbial species such as *c_Bacilli, o_Lactobacillales, f_Lachnospiraceae, s_uncultured_bacterium_g_Lactobacillus, g_Lactobacillus, f_Bacteroidaceae, g_Bacteroides, and s_uncultured_bacterium_g_Bacteroides, s_Bacteroides_acidifaciens.* An increase in the proportions of three types of immune cells might associated with the above dominant bacterial species.

**Conclusion:** Kaempferol can inhibit xenograft lung cancer models. Such inhibition effect might come from the activation of T cells, NK cells, and other immune cells which are modulated by the gut microbiota.

## Introduction

Lung cancer is currently the second most common malignant tumor in the world and exhibits the highest mortality rate 1. According to GLOBOCAN data, the estimated number of new cases of lung cancer was 2.207 million, and the number of deaths was 1.796 million 2. In China, the incidence and mortality of lung cancer rank first among all malignant tumors, with 828,000 new cases accounting for 24.6% of all new cancer cases and 657,000 deaths accounting for 29.71% of all cancer deaths in 2020 3. Although Traditional Chinese Medicine (TCM) has shown benefits in improving survival in lung cancer patients when used alone or in combination with multidisciplinary treatments 4, the mechanisms underlying the effects remain unclear. It is therefore necessary to explore novel biomarkers and to further elucidate the mechanistic aspects of lung cancer treatment to provide new strategies and methods to optimize tumor therapy.

The gut microbiota is a collective term for the various microorganisms that reside in the human intestine—including bacteria, fungi, and viruses—and it plays an important role in maintaining normal physiological and immune function in the human body 5. The foods, drugs, and herbal supplements ingested by humans are digested and absorbed through the intestines and can influence the onset, development, treatment, and prognosis of various diseases (including cancer) by affecting the gut microbiota 5. Recent studies have revealed that changes in the gut microbiota are closely related to chemotherapy, radiotherapy, targeted therapy, immunotherapy, and other cancer treatments. The composition of the gut microbiota and its changes during cancer treatments may affect the treatment effects, thus serving as a target for improving cancer-treatment effects and reducing toxicity. Moreover, it can provide novel strategies for the prevention, diagnosis, and treatment of cancer 6-8.

Although the gut and lungs are distant anatomically, they form the gut-lung axis through complex bidirectional lymphatic and blood communications 9. This interaction can affect energy metabolism, innate and adaptive immune function locally and systemically, furthermore distally arising cancers may also be modulated by barrier disruption and the translocation of microorganisms and metabolites, and by the interactions between immune cells and microbial products 8. According to the “gut-lung axis" theory, gut microbes can maintain the balance between tumor-promoting inflammatory responses and antitumor immune responses. Both bacteria and their metabolites can exert systemic effects on the human body 10. There are two primary roles postulated for the immune system in the gut-lung axis 10: (1) Immune cells and antibodies are stimulated by the intestinal flora and migrate to the lungs, the intestinal submucosa, or gut-associated lymphoid tissues that contain a large number of macrophages and other immune cells, *i.e.*, the intestinal flora influence the immune response of the lungs through mucosal immunity 11,12. Pathogens that enter the gut in a healthy state stimulate the conversion of interleukins (pro-IL-1β and pro-IL-18) in gut-associated lymphoid tissue into active inflammatory factors and inhibit the body's innate ability to produce IL-10 and other anti-inflammatory molecules. Mature memory T cells and plasmablasts migrate to mucosal lymphoid tissues that include the pulmonary bronchial epithelium through a homing mechanism 13, thereby improving the ability to rely on inducible cell lines (Th1 and Th2) and targeting the immune responses of pathogenic bacteria in the lungs 14. (2) The translocated gut flora and their products influence the immune responses of the lung. Gut microbiota induces the generation of CD4+ T cells with autoantigens, and, in addition, Th17 and memory Th1 cells induced by gut flora products or pattern-recognition receptor ligands may be preferentially enriched in the inflammatory tumor microenvironment (TME) 15. The long-term effect of the flora on the immune system is hypothesized to be due to the phenomenon of antigen mimicry or cross-reaction: the flora crosses the intestinal barrier and stimulates T cells, thereby promoting immune system reactivity and antitumor response, and is an agent of immunosurveillance. After crossing the intestinal barrier, bacterial flora interacts with pattern-recognition receptors and stimulates the intestinal lymphatic system to produce various cytokines and interferons, thus inducing pro-inflammatory, immunostimulatory, or immunosuppressive responses 16.

Apart from the direct effects of the gut-lung axis, the gut microbiota and cytokines produced by the gut-associated peripheral microbiota also regulate the immune responses of distant organs 17. According to the "gut-lymph" theory 18, some protein fragments from live bacteria, cell wall debris, or dead bacteria that are not blocked by the first line of defense are capable of escaping via cytokines and chemokines produced by the gut lymph. These fragments are then transferred to gut-associated lymphoid tissues by antigen-presenting cells, enter the lymphatic circulation through chyle pools, and eventually reach the pulmonary mucosal lymph nodes where they activate dendritic cells and macrophages and initiate and differentiate B and T cell progenitors—thereby affecting the immune responses of the lungs. Some metabolites such as short-chain fatty acids may be potential mediators of distant gut effects, and directly or indirectly affect target organs by activating the gut/circulatory immune system 19-22. However, the relationships among gut microbiota, immunity, and lung cancer remain arcane, and additional research is sorely needed.

Kaempferol (C15H10O6), a flavonoid compound, has been widely found in edible plants and herbs. Kaempferol and its glycosylated derivatives have been shown to exert antioxidant, anti-inflammatory, antibacterial, anticancer, cardioprotective, neuroprotective, antidiabetic, anti-osteoporotic, anxiolytic, analgesic, and antiallergic effects 23. Investigators have recently shown that kaempferol possesses great potential in antitumor therapy and that it can exert its effects against various cancers, including non-small cell lung cancer 24. Kaempferol regulates the AKT/PI3K- and ERK-signaling pathways; inhibits the phosphorylation of TIMP2 and MMP2; downregulates the protein expression of Bcl-2, cyclin D1, and claudin-2; inhibits STAT3 factor binding; upregulates PTEN, Bax, miR-340, Fas, cleaved-caspases 3, 8, and 9, and cleaved-PARP; promotes cellular apoptosis; reduces cell migratory and invasive capabilities; and arrests the cell cycle at the G2/M phase 25-27. However, how kaempferol regulates the gut microbiota to exert therapeutic effects are still not clear. Therefore, we explored the biological mechanisms of kaempferol for the treatment of lung cancer through drug intervention in subcutaneous xenograft mouse models and high-throughput 16S rRNA sequencing of the gut.

## Materials and Methods

### Cell culture

We purchased the murine LLC cell line from the Cell Bank/Stem Cell Bank of the China Center for Type Culture Collection. The cells were cultured in a humidified atmosphere of 37°C and 5% CO_2_ in RPMI-1640 (Gibco, Billings, MT, United States) supplemented with 10% fetal bovine serum (Gibco) and antibiotics (100 U/mL penicillin and 100 μg/mL streptomycin). Cells continued to be cultured under conditions identical to those above and passed to the second generation before further experimentation.

### *In vivo* xenografts and drug treatment

Twenty-four 10-week-old male BALB/cByJ mice were purchased from Shanghai SLAC Laboratory Animal Co. Ltd. After a week of acclimatization, logarithmically grown Lewis Lung Carcinoma (LLC) cells (approximately 5×10^5^ cells) were inoculated into the right lower back of twelve of these BALB/c mice to establish xenograft lung cancer models. The 12 tumor-bearing mice were divided into the LLC group and the LLC+Kaem group, while the remaining normal mice were split into the BC group and the BC+Kaem group. All groups were fed a normal diet. The BC+Kaem and LLC+Kaem groups were treated with Kaempferol via gavage, while the BC and LLC groups were treated with PBS. In relevant studies of kaempferol, doses of 20 mg/kg, 50 mg/kg, and 100 mg/kg exert inhibition of tumor 28-30. Many experiments have demonstrated that a high dose of kaempferol does not produce cytotoxicity but plays a protective role 31,32. Therefore, for efficacy and safety reasons, the administration dose of kaempferol was 50 mg/kg by gavage. After 21 days, the mice were euthanized, and the tumors were surgically removed. Tumor weight (g) and volume (mm^3^) were measured, with the volume calculated by applying the formula: 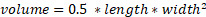. The study was approved by the Animal Welfare and Ethics Committee of Shanghai University of Traditional Chinese Medicine (No. PZSHUTCM210514013), and all experiments were performed in compliance with the "Regulations for the Administration of Experimental Animals" issued by the State Science and Technology Commission.

### Hematoxylin and eosin (H&E) staining

Tissue samples were fixed in 4% paraformaldehyde, dehydrated, and embedded in paraffin; and we cut sections at 4 μm using a microtome and placed them on glass slides. The slides containing sections were dewaxed with xylene and then rehydrated through a gradient series of ethanol concentrations (100%, 90%, 80%). The slides were then stained with hematoxylin for five minutes at 25°C, followed by differentiation in 1% hydrochloric acid in ethanol for 30 seconds, and immersion in 0.6% ammonia water for one minute to turn the nuclei blue. The slides were ultimately washed with distilled water for five minutes, stained with eosin for two minutes at room temperature, and washed with distilled water for two minutes. Slides were dehydrated with a gradient series of ethanol concentrations (75%, 80%, 95%, 100%) and cleared with xylene for two minutes. The sections were then mounted with neutral gum, examined under a microscope, and images were captured and analyzed.

### Flow Cytometry

Mononuclear cells from peripheral blood were isolated from each group of mice, and the cellular clusters were collected and adjusted to a density of 1×10^6^ cells/mL in 0.2 mL of Dulbecco's Phosphate-Buffered Saline (DPBS) containing 10% Bovine Serum Albumin (BSA). After adding the primary antibody, the cells were mixed and incubated at 4°C for 30 min in the dark. We then compared cellular staining with the isotype control antibodies (mouse IgG1-FITC and mouse IgG1-PE; Invitrogen, eBioscience, Shanghai, China) to correct for non-specific binding. Antibody staining was evaluated with flow cytometry using FACS Aria (Quanta SC, Beckman Coulter, Inc.).

### 16S rRNA sequencing analysis of gut microbiota

Fresh fecal samples were collected during the last five days of the experiment to analyze the gut microbiota as previously described 33, and bacterial genomic DNA was extracted from frozen samples stored at -80°C. The V3 and V4 regions of the 16S rRNA gene were amplified using specific primers (F primer, 5'-ACTCCTACGGGAGGCAGCA-3'; R primer, 5'-GGACTACHVGGGTWTCTAAT-3'), and sequenced using high-throughput Illumina sequencing at Biomarker Technologies (Beijing, China). The original paired-end reads of the DNA fragments were merged using FLASH32, and unique barcodes were assigned to each sample. Tags were clustered into operational taxonomic units (OTUs) using the UCLUST tool in QIIME (v1.8.0) and based on 97% sequence identity. α-diversity was evaluated using mother software (v1.30), and diversity indices were compared among samples by standardizing the sequence numbers. We constructed rank, rarefaction, and Shannon curves; and Shannon, Chao, Simpson, and abundance-based coverage estimators were calculated for OTUs. For β-diversity analysis, key OTUs were identified using heatmaps of RDA-identified, principal coordinate analysis (PCoA), non-metric multidimensional scaling (NMDS), and unweighted pair-group method with arithmetic mean (UPGMA) in Quantitative Insights into Microbial Ecology (QIIME, v1.8.0). Linear discriminant analysis (LDA) effect size (LEfSe) was used to quantitatively analyze biomarkers. Abundant taxa with significant differences were identified using LEfSe (LDA score > 4.0), non-parametric Kruskal-Wallis and rank-sum tests, and unpaired Wilcoxon rank-sum tests.

### Statistical analysis

Each experiment was performed at least three times and GraphPad Prism 9 was used for statistical analysis. Data are presented as mean±standard error. We compared differences using Student's t-test and one-way ANOVA with a significance level of *p* < 0.05.

## Results

### Kaempferol inhibits the activity of xenograft lung cancer models *in vivo*

**Figure 1:**
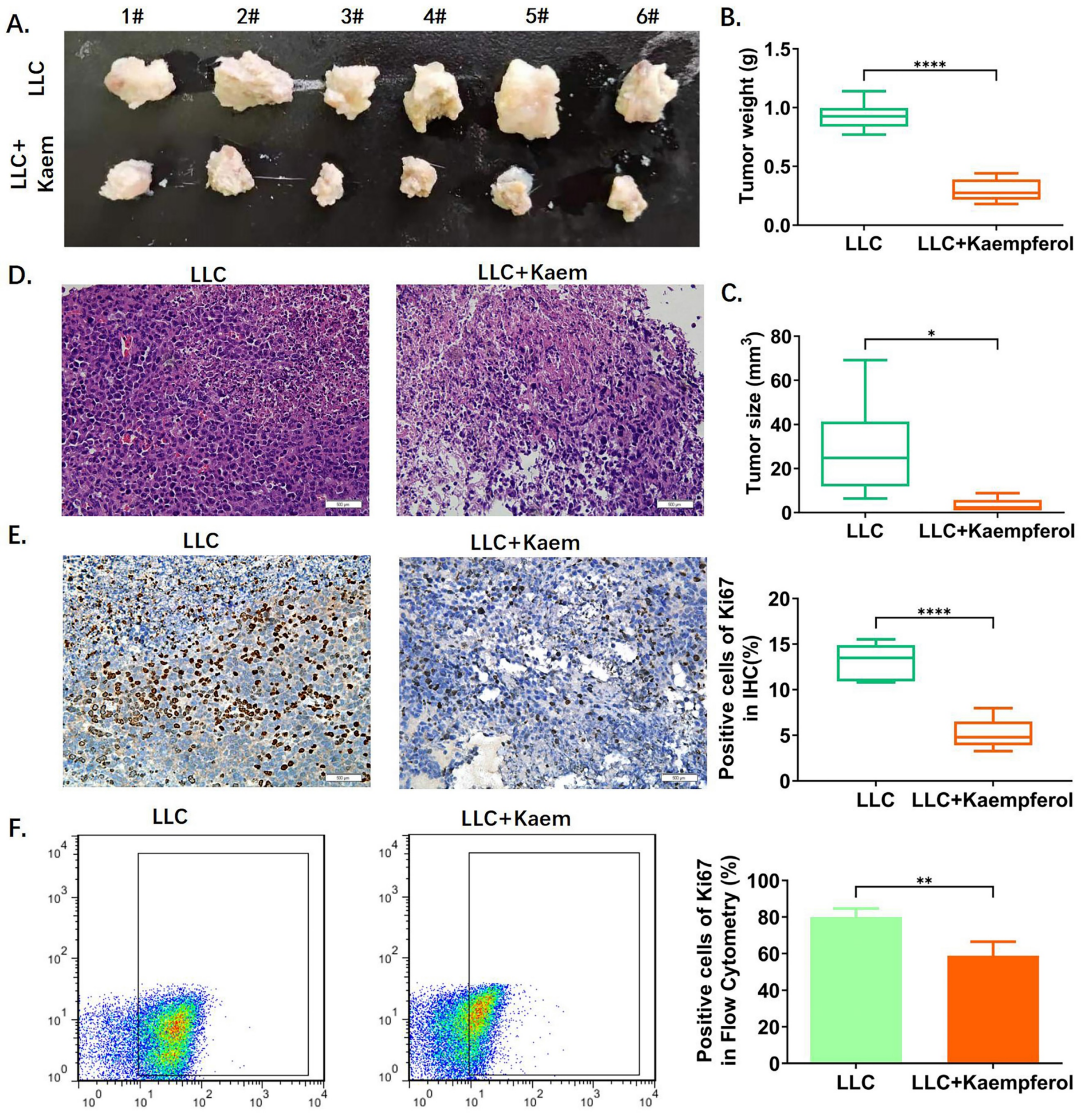
** Kaempferol inhibits the activity of xenograft lung cancer *in vivo*.** (A) Tumor morphology. (B), (C) Tumor weight and volume. ****p < 0.0001***p < 0.001; **p < 0.01; *p < 0.05; n = 6; *t*-test. (D) Hematoxylin-eosin staining of tumor tissues. (E) Immunohistochemical staining of Ki67 and its quantification. The ratios of ki67 among different groups. ****p < 0.0001***p < 0.001; **p < 0.01; *p < 0.05; n = 6; *t*-test. (F) Flow cytometry of Ki67 in tumor tissues. *p < 0.0001 vs. LLC; n = 6; *t*-test.The ratios of ki67 among different groups. **p < 0.01; *p < 0.05; n = 4; *t*-test. The above experiments were obtained from xenograft lung cancer samples of mice.

Analysis of Balb/c tumor-bearing mice showed that the tumor morphology in the LLC+Kaem group was smaller compared to the LLC group (Figure 1A). Tumor weight and volume in the kaempferol group were also significantly lower than in the control group (p < 0.01) (Figure 1B, C). Further, H&E staining of tumors (Figure 1D) showed that the results between treatment and control groups were consistent with the pathological characteristics of lung cancer, but the malignancy manifested in the treatment group was lower. Immunohistochemical results showed that compared with the control group, the proportion of Ki67-positive cells in the LLC+Kaem group significantly declined (*p* < 0.0001) (Figure 1E, F). These results suggest that kaempferol significantly inhibits the proliferation of LLC subcutaneous xenograft mice.

### Kaempferol stimulates immune cell activity

Flow cytometric analysis demonstrated significant changes in immune cell profiles following Kaempferol intervention. Compared to the BC group, the BC+Kaem group showed significantly higher proportions of CD8+/INFγ+, CD49b+/CD107a+, and CD68+/INFγ+ cells in the peripheral blood, while these proportions were lower in the LLC group. In comparison with the LLC group, the LLC+Kaem group exhibited increased levels of these cell populations. This indicates that Kaempferol intervention activates cytotoxic T cells, natural killer cells, and macrophages in both normal mice and those with LLC subcutaneous xenograft tumors. Additionally, Ki67 staining results revealed a decreased number of Ki67-positive cells in the LLC+Kaempferol group. Together with previous immunohistochemical findings, this suggests that Kaempferol suppresses tumor growth, influencing both the phenotype and the tumor microenvironment.

### Regulation of gut microbiota distribution and metabolism in LLC subcutaneous xenograft mode mice by kaempferol

**Figure 3:**
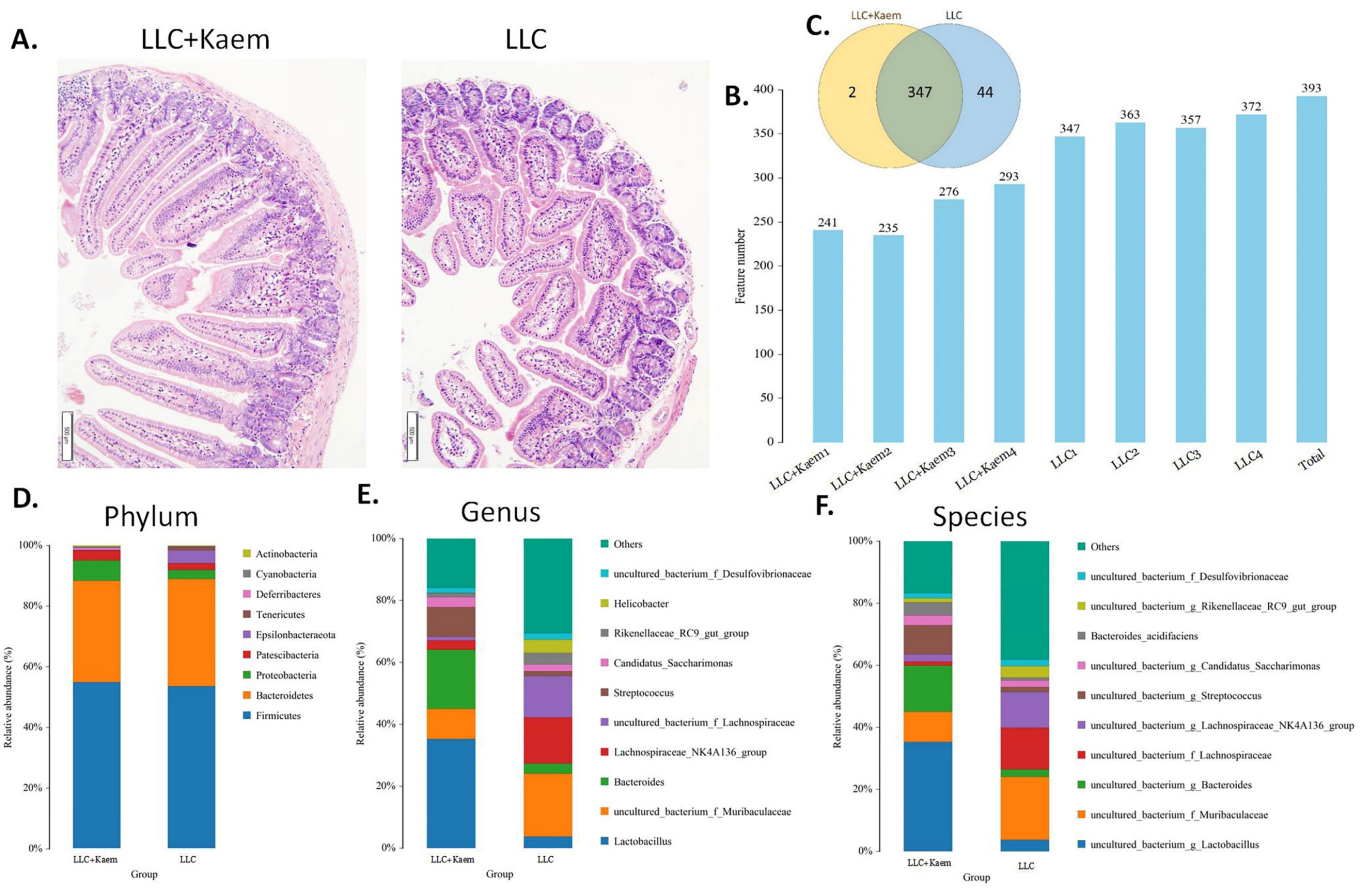
** Histomorphology of the colon and Analysis of OTUs.** (A) Histomorphology of the colon in LLC-bearing mice. Samples were obtained from the mouse colon. (B) Numbers of OTUs. (C) Venn diagram depicting 347 OTUs as common between the groups. (D) Gut-microbiota species distribution at the phylum level. (E) Gut-microbiota species distribution at the genus level. (F) Gut-microbiota species distribution at the species level. Analysis of OTU Samples were obtained from the mouse intestinal contents.

Histopathological evaluation of the colon revealed that kaempferol treatment could reduce the infiltration of inflammatory cells in the colon and make the intestinal mucosa more complete (Figure 3A). Intestinal contents of the LLC+Kaem group and LLC group were collected to evaluate the composition and specific distributions of gut microbiota by sequencing 16S rRNA v3+v4 regions of fresh fecal samples. A total of 640,247 reads were obtained from the eight samples sequenced, and 637,278 clean reads were produced after quality control and splicing of paired-end reads, with each sample generating a minimum of 79,459 clean reads and a mean of 79,660 clean reads. Using UCLUST software in QIIME (version 1.8.0), tags were clustered into OTUs based on 97% sequence identity, and we noted that the numbers of OTUs in the LLC+Kaem and LLC groups differed significantly (Figure 3B). There were 347 overlapping OTUs between the two groups, with 44 unique OTUs in the LLC group and two unique OTUs in the LLC+Kaem group (Figure 3C). By comparing the representative sequences of the OTUs with microbial reference databases, each OTU was classified into a species. The composition of the community was then calculated for each sample. Different levels of classification (kingdom, phylum, class, order, family, genus, and species) were used to generate species-abundance tables using QIIME software. We then calculated the community structure of samples at different classification levels using R tools. Phylum-level analysis showed that the relative abundances of *Patescibacteria* and *Proteobacteria* in intestinal contents from LLC+Kaem mice significantly increased compared to those in the LLC group, while the relative abundance of *Epsilonbacteraeota* significantly decreased. Genus-level analysis showed that the relative abundances of *Bacteroides* and *Lactobacillus* in LLC+Kaem mice significantly increased compared to that in the LLC group, while the relative abundances of *Others*, Lachnospiraceae_NK4A136_group,* uncultured_bacterium_f_Lachnospiraceae,* and* uncultured_bacterium_f_Muribaculaceae* significantly declined.

**Figure 4:**
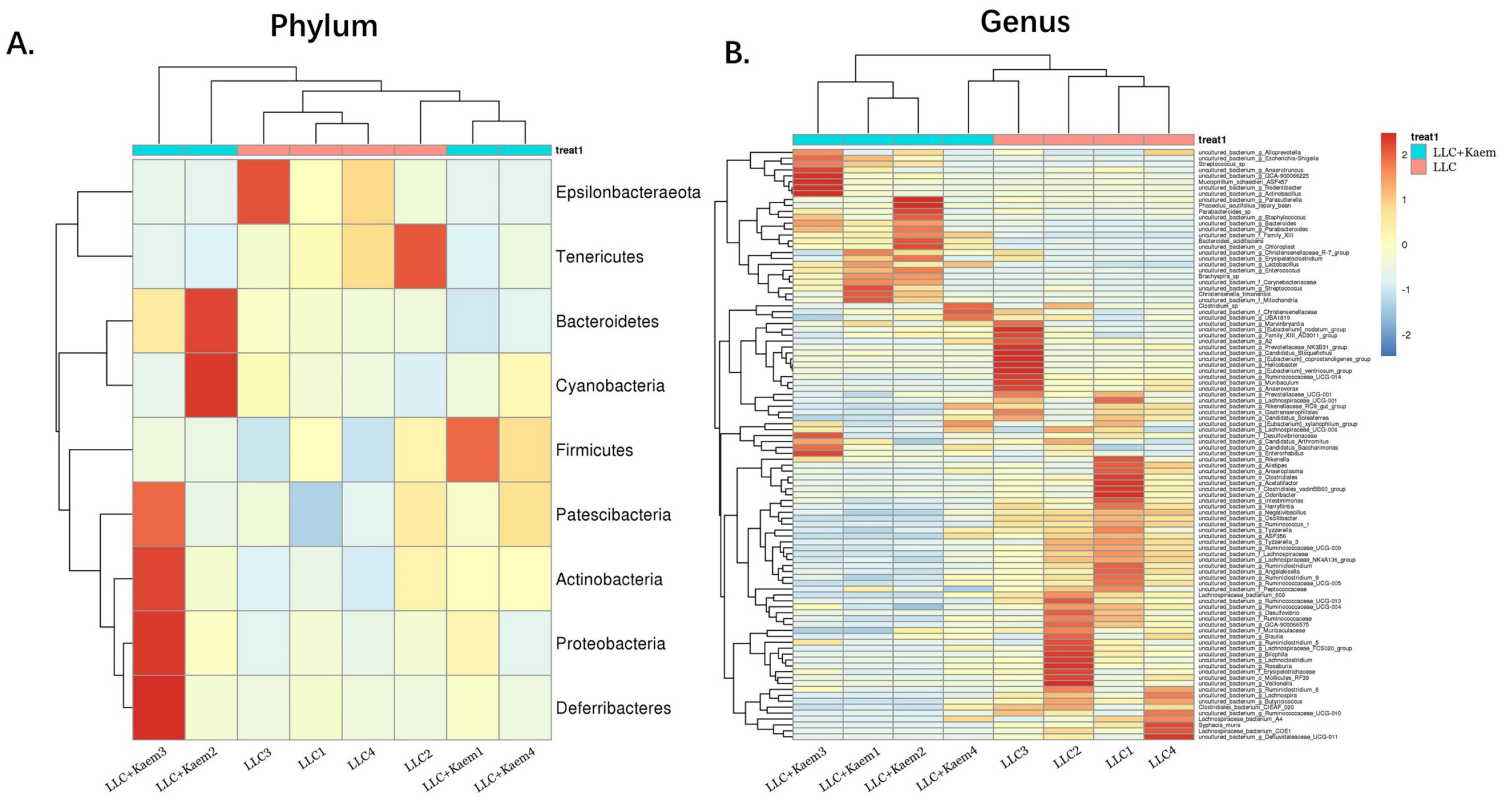
** Heatmap of species abundances.** (A) Phylum-level analysis: the relative abundances of Bacterokletes, Cyanobacteria, Firmicutes, Patescibacteria, Actinobacteria, Proteobacteria, and Deterribacteres microbes with kaempferol treatment. (B) Genus-level analysis. All the resulting samples were derived from mouse intestinal contents.

**Figure 5:**
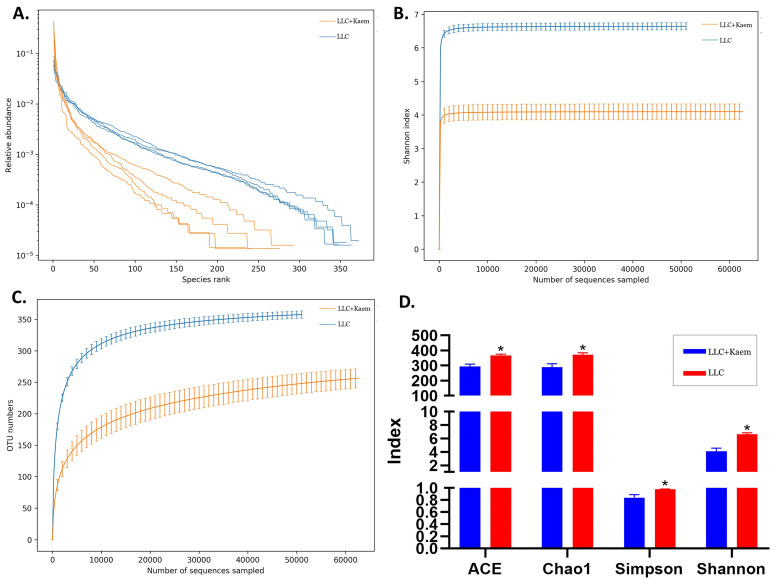
** α-Diversity analyses.** (A) Rank abundance curve. (B) rarefaction curve. (C) Shannon index curve. (D) alpha-diversity analysis. All the resulting samples were derived from mouse intestinal contents.

**Figure 6:**
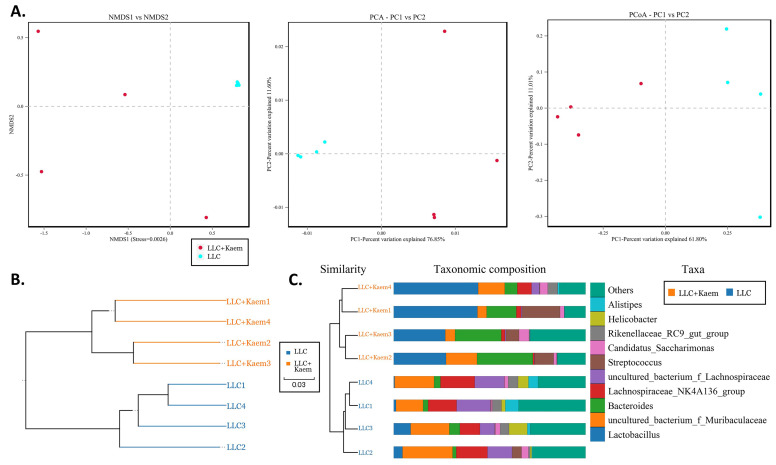
** β-diversity analyses.** analysis(A) Results of PCA, PCoA, and NMDS analysis. (B) Unweighted-sample, pair-group method with an arithmetic-mean clustering tree. (C) Combined illustration of clustering tree and histogram. All the resulting samples were derived from mouse intestinal contents.

**Figure 7:**
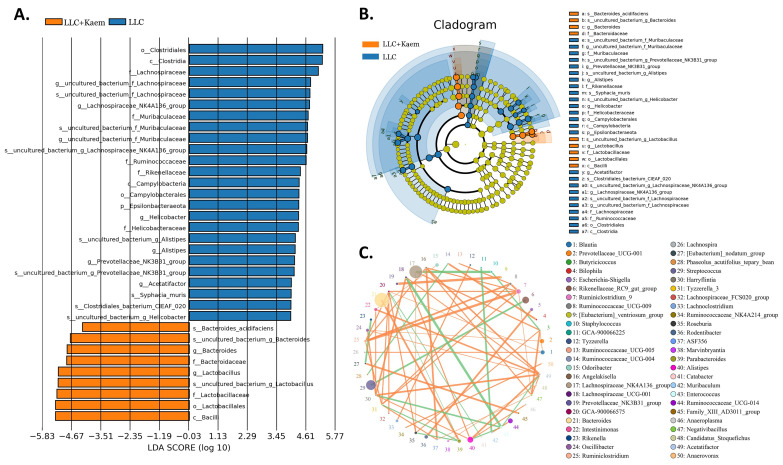
** Significant-difference analysis between groups.** (A) Value-distribution histogram of line-discriminant analysis effect size (LEfSe). (B) Results of species annotation were visualized. (C) Species network at the genus level. All the resulting samples were derived from mouse intestinal contents.

**Figure 8:**
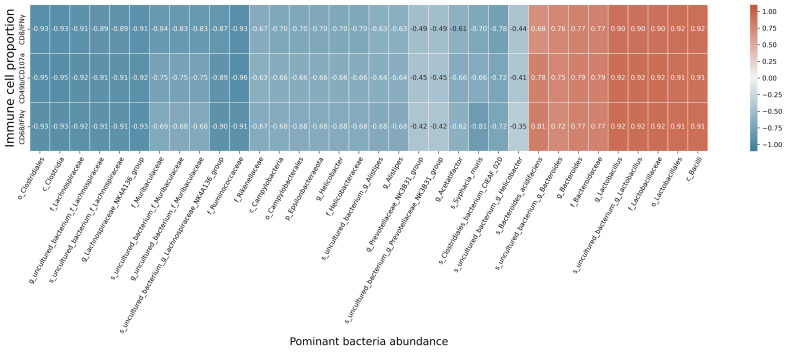
** Correlation analysis.** Correlation analysis between the abundance concentrations of strains screened by LDA and the ratios of CD8+/INFγ+, CD68+/INFγ+, and CD49b+/CD107a+ cells from flow-cytometric analysis.

At the species level, the relative abundances of *uncultured_bacterium_g_Bacteroides* and *uncultured_bacterium_f_Lactobacillus* in the intestinal contents of LLC+Kaem mice were significantly elevated compared to the LLC group, while the relative abundance of *uncultured_bacterium_f_Muribaculaceae* was significantly reduced (Figure 3D-F). Microbial-diversity-clustering analysis showed that the microbial diversity of LLC+Kaem mice gut microbiota was principally derived from *Bacterokletes, Cyanobacteria, Firmicutes, Patescibacteria, Actinobacteria, Proteobacteria, Deterribacteres microbes* (increased in number), and *Epsilonbacteraeota, Tenericutes* microbes (decreased in number) (Figure 4). Alpha-diversity analysis revealed that both groups exhibited flat rank abundance curves, indicating a high level of evenness in the composition of species (Figure 5A). A smooth rarefaction curve indicated that the sample sequences were sufficient for data analysis (Figure 5B). The Shannon index lines of the two groups were flat, indicating that the sequencing data volume was large enough and that the OTU types did not show a commensurate rise with increasing sequencing volume (Figure 5C). Beta-diversity analysis showed that the inter-group differences in microbial communities could be analyzed using the Bray-Curtis algorithm. This analysis mainly included PCoA, principal component analysis (PCA), and NMDS. The analysis revealed significant differences in the gut microbial composition between the LLC+Kaem and LLC groups, with distinct clustering observed in their microbial communities. (Figure 6A). The results of the UPGMA sample hierarchical-clustering analysis suggested that the homology of the intestinal microbiota between the LLC+Kaem and LLC groups was low and that there was no close genetic background (Figure 6B, C). In addition, LEfSe was used to identify high-dimensional biomarkers in the intestinal microbiota of each group, with the LDA score set to 4.0, and LDA scores greater than 4 for different species were considered important biomarkers. As shown by the cladogram analysis and the distribution of LDA scores, the number of microorganisms in the LLC+Kaem group—such as *c__Bacilli, o_Lactobacillales, f__Lachnospiraceae, s__uncultured_bacterium_g_Lactobacillus, g__Lactobacillus, f__Bacteroidaceae, g_Bacteroides, s__uncultured_bacterium_g_Bacteroides, s__Bacteroides_acidifaciens,* and other families—increased significantly (Figure 7A-7C). Therefore, these microbial communities might comprise uniquely advantageous strains for treatment. Correlation analysis between the abundance concentration of strains screened by LDA and the results of the flow-cytometric analysis revealed that the abundances of dominant strains were positively correlated with increased ratios of CD8+/INFγ+, CD68+/INFγ+, and CD49b+/CD107a+ cells (Figure 8).

### Kaempferol regulates the differential expression of functional genes and metabolic signaling pathways in the gut microbiota of LLC mouse models

**Figure 9:**
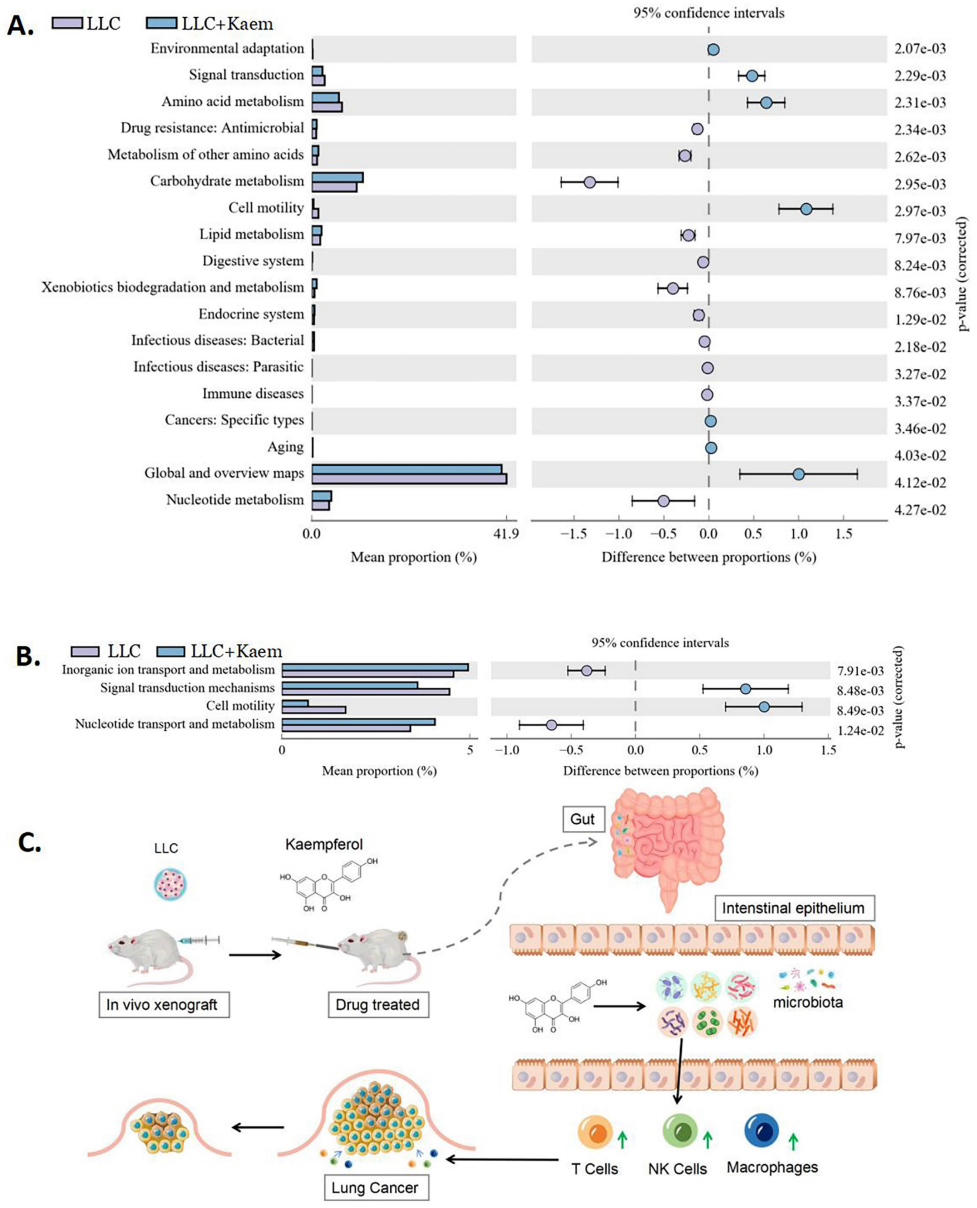
** Metabolic-signaling pathways and protein differences in pulmonary microbiota.** (A) Results of Kyoto Encyclopedia of Genes and Genomes (KEGG) metabolic-pathway analysis. (B) Clusters of orthologous groups following the protein analysis of distributions and abundances of homologous protein clusters in pulmonary microbiota. (C) Mechanistic diagram: Kaempferol activates immune cells to treat lung cancer by modulating gut microbiota.

Differential analysis of functional genes in the microbial community among different groups can reveal the changes in the metabolic functions of samples in response to environmental changes. From KEGG-based pathway analysis, we determined that the microbial communities in the intestines of mice in the LLC+Kaem group exhibited elevated metabolic pathways such as Carbohydrate metabolism (Metabolism), Lipid metabolism (Metabolism), and Nucleotide metabolism (Metabolism) while showing reduced metabolic pathways such as Signal transduction (Environmental Information Processing), Amino acid metabolism (Metabolism), and Global and overview maps (Metabolism), compared to the LLC group (Figure 9A). Analysis using clusters of orthologous groups (COGs) of proteins revealed the distributions and abundances of homologous protein clusters in the microbial community. Our results showed that the abundances of Inorganic ion transport and metabolism (Metabolism) and Nucleotide transport and metabolism (Metabolism)-related proteins in the gut microbiota of mice in the LLC+Kaem group significantly increased, while the abundances of Signal transduction mechanisms (Cellular Processes and Signaling) and Cell motility (Cellular Processes and Signaling)-related proteins significantly decreased, compared to the LLC group (Figure 9B).

## Discussion

In this study, we aimed to explore three key aspects: 1) The inhibitory effects of kaempferol on xenograft lung cancer models, 2) its impact on immune cell responses in these models, and 3) its regulatory influence on the gut microbiota. Currently, previous studies have reported the effect of kaempferol against lung cancer *in vitro*, it can block TGF-β1-induced EMT and cancer cell migration by inhibiting Akt1-mediated phosphorylation of the Thr179 residue of Smad3 34.In our study, we first proved the therapeutic effect of kaempferol on xenograft lung cancer models in the *in vivo* experiment, and found that the volume and weight of tumors in mice treated with kaempferol were significantly reduced; and the results of pathological sections showed that the degree of malignancy was low. This evidence shows that kaempferol can reduce the tumor size of tumor-bearing mice and inhibit the proliferation of LLC cells. Investigators recently showed that kaempferol exerted its antitumor effects from multiple dimensions, including signal transduction, cell cycle, energy metabolism, anti-angiogenesis, anti-metastasis, and anti-inflammation 35. However, there is no extant evidence that kaempferol directly stimulates immune cells. So we conducted flow cytometric analysis on peripheral blood cells of the mice, determining that Kaempferol significantly activates cytotoxic T cells, natural killer cells, and macrophages in both normal and LLC subcutaneously xenografted mice. This suggests that post-Kaempferol treatment, tumor-bearing mice exhibited enhanced immune activity. Importantly, this direct activation effect is not attributed to apoptosis of tumor cells or the release of cytokines within the tumor tissue.

We administered kaempferol orally by gavage and absorbed it after digestion in the gastrointestinal tract. Given the large numbers of macrophages and other immune cells in the submucosal layer of the intestine or intestine-related lymphoid tissues and the maturation of T cells on the mucosa 36, we speculated that the gut microbiota might play an active role in kaempferol's effects. We evaluated the composition and specific distribution of gut microbiota in fresh fecal samples from different groups of mice using 16S rRNA v3+v4 region sequencing. LDA analysis of the sequencing results showed that the abundance of certain microbes such as *c__Bacilli, o__Lactobacillales, f__Lachnospiraceae, s__uncultured_bacterium_g_Lactobacillus, g__Lactobacillus, f__Bacteroidaceae, g_Bacteroides, s__uncultured_bacterium_g_Bacteroides,* and *s__Bacteroides_acidifacien* increased significantly in the gut microbiota of mice treated with kaempferol, indicating their potential advantages in treating tumors. Furthermore, the correlation between the dominant bacterial species and the flow-cytometric results revealed that the abundance of these bacteria was positively correlated with the activity of cytotoxic T cells, natural killer cells, and macrophages in mice. Recent research has also shown that most of these bacterial species can regulate the immune system through specific mechanisms. For example, the metabolites of Bifidobacteriaceae contain a large number of short-chain fatty acids (SCFAs) such as acetic acid, propionic acid, and butyric acid; and the intestinal microbial metabolite butyric acid can promote key transcriptional regulation in the process of immune cell differentiation and activation 37. The expression of factor ID2 regulates the IL-12-signalling pathway and enhances the immune responses of CD8+ T cells to improve the efficacy of antitumor drugs 37. As a probiotic, *Bacteroides acidifaciens* regulates metabolites and enhances host immunity, promotes the accumulation of Th1 and Th17 cells in the gut, and facilitates the polarization of helper T cells 38,39.

*Acidophilus* is also one of the main bacteria responsible for the production of immunoglobulin IgA by increasing the number of IgA+ B cells and activating B cells in the large intestine 40. *Lactobacillus* belongs to the Firmicutes, which also includes *Lactobacillus* as a natural immunobiological agent with favorable immunoregulatory function. Some studies have shown that lactic acid bacteria, as one of the most critical probiotics in tumor research, are expected to comprise an effective adjuvant in tumor therapy. For example, Zhang et al. 41 found in their *in vivo* studies that *Lactobacillus casei* inhibited the proliferation of A549 lung cancer cells, and Matsuzaki T et al. 42 found that the same species significantly improved the antitumor activity in mouse models of lung cancer. Le Noci et al. 43 demonstrated that aerosol inhalation of *Lactobacillus rhamnosus* inhibited lung cancer growth by promoting local immunity. Lachnospiraceae can produce a large number of short-chain fatty acids that regulate the body's immune response 44. Guo et al. 45 determined that Lachnospiraceae also exerted a protective effect against radiation damage and that its significantly increased abundance in mice showed longer survival after radiotherapy. Gut flora can activate immune cells in the tumor immune microenvironment to portray an antitumor role, and it is closely associated with T lymphocytes, natural killer (NK) cells, macrophages, and other immune cells 46. Gut microbiota can modulate CD4+ T cell responses to improve immunosurveillance and enhance therapeutic efficacy. In one study, 11 bacterial strains were found to enhance the expression of MHC class I molecules, activate IFN-γ+ CD8 T cells and inhibit the growth of colorectal cancer 47.

Microbial metabolites such as SCFAs, secondary bile acids, polyamines, and vitamins are also capable of influencing the development of cancer and the efficacy of antitumor treatment 48. For example, short-chain fatty acids (particularly propionate and butyrate) are beneficial for the differentiation and accumulation of Treg cells, and thereby mediate anti-inflammatory effects; secondary bile acids can directly or indirectly affect the composition of the intestinal microbiota through immune activation and vitamin B6 can promote immune monitoring in non-small cell lung cancer chemotherapy 49,50. Li et al. 29 found that lactic acid bacteria as well as short-chain fatty acids were mainly present in the intestine of colorectal mice treated with kaempferol, and kaempferol could reduce the tumor burden in mice by regulating bile acid signaling and gut microbial homeostasis. Based on the analysis of metabolic pathways in KEGG database, we further hypothesize that kaempferol promotes pathways such as Carbohydrate metabolism (Metabolism), Lipid metabolism (Metabolism), and Nucleotide metabolism (Metabolism) while reducing metabolic pathways such as Signal transduction (Environmental Information Processing), Amino acid metabolism (Metabolism), and Global and overview maps (Metabolism) to activate immune cells.

Our results suggest that the potential mechanism by which kaempferol inhibits the progression of xenograft lung cancer in mice might involve the activation of immune cells, possibly through the modulation of gut bacterial microecology. (Figure 9C). However, the intricate relationship between these factors still needs further investigation.

## Conclusions

In this study, we have investigated the effects of kaempferol in a mouse lung cancer xenograft model. We assessed the changes in the peripheral blood immune cells and utilized 16S rRNA sequencing of the intestinal contents to explore potential mechanisms underlying the treatment's efficacy. We found that kaempferol treatment reduced the progression of xenograft lung cancer in mice, induced the activation of immune cells in peripheral blood, and regulated the distribution of gut microbiota. These observations suggest that kaempferol might activate T cells, NK cells, and other immune cells through gut microbiota modulation, which potentially elicits the beneficial immune response in lung cancer treatment.

## Figures and Tables

**Figure 2:**
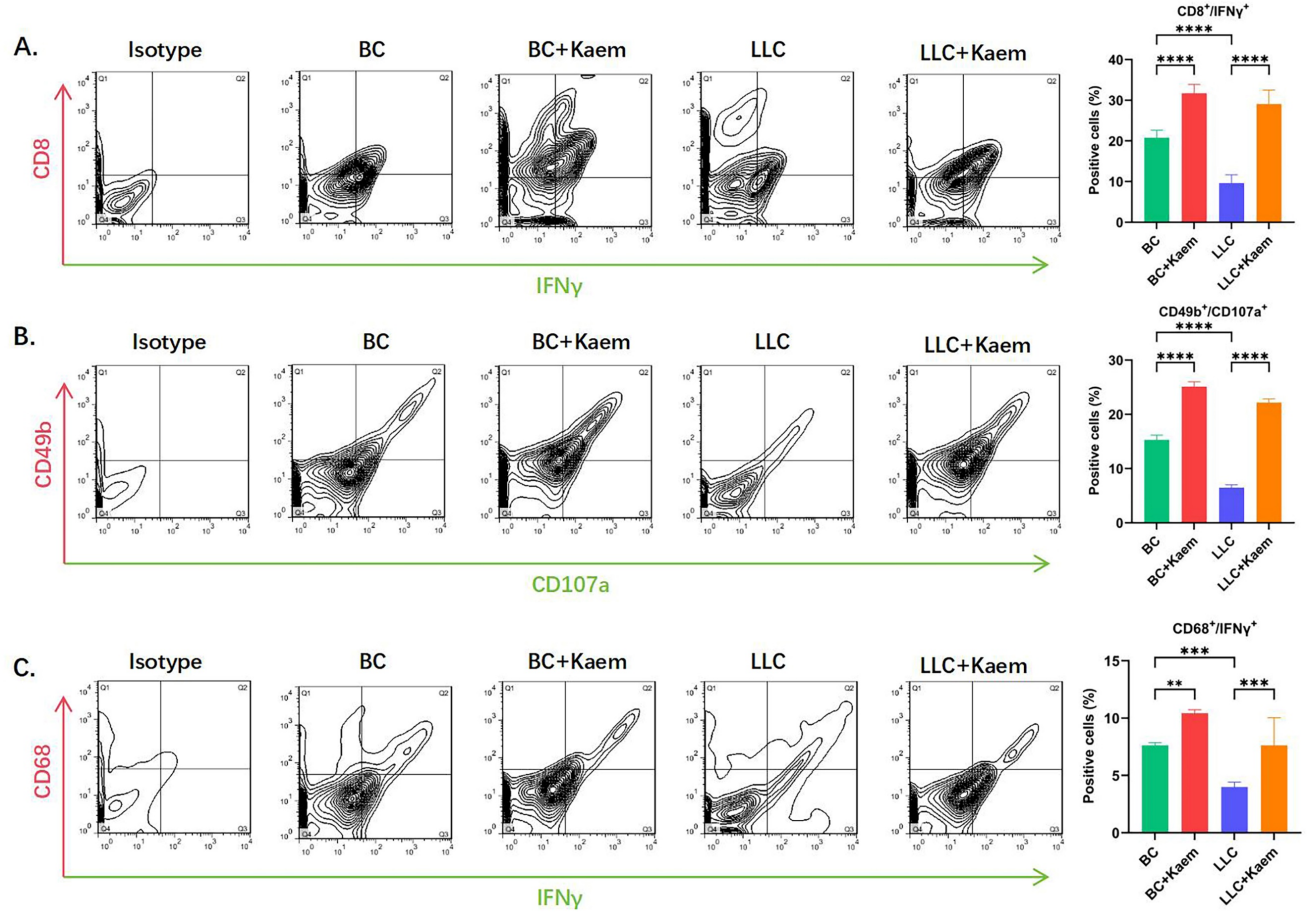
** Flow-cytometric analysis.** (A)The ratios of CD8+/INFγ+ cells among different groups. ****p < 0.0001***p < 0.001; **p < 0.01; *p < 0.05; n = 6; one-way ANOVA. (B) The ratios of CD49b+/CD107a+ cells among different groups. ****p < 0.0001***p < 0.001; **p < 0.01; *p < 0.05; n = 6; one-way ANOVA. (C) The ratios of CD68+/INFγ+ cells among different groups. ****p < 0.0001***p < 0.001; **p < 0.01; *p < 0.05; n = 6; one-way ANOVA. The above experiments were obtained from the peripheral blood of the mice.

## Abbreviations

TCM: Traditional Chinese Medicine
PBS: phosphate buffered saline
LLC: Lewis lung carcinoma
DPBS: Dulbecco's Phosphate-Buffered Saline
BSA: Bovine Serum Albumin
TME: tumor microenvironment
OTUs: operational taxonomic units
PCoA: principal coordinate analysis
PCA: principal component analysis
NMDS: non-metric multi-dimensional scaling
UPGM: Unweighted pair-group method with arithmetic mean
LDA: Linear discriminant analysis
LEfSe: Linear discriminant analysis effect size
Kaem: kaempferol
SCFAs: short-chain fatty acids
KEGG: Kyoto Encyclopedia of Genes and Genomes
IHC: Immunohistochemistry
